# Topical anti-inflammatory activity of palmitoleic acid improves wound healing

**DOI:** 10.1371/journal.pone.0205338

**Published:** 2018-10-11

**Authors:** Eleine Weimann, Maysa Braga Barros Silva, Gilson Masahiro Murata, Jose Ricardo Bortolon, Alexandre Dermargos, Rui Curi, Elaine Hatanaka

**Affiliations:** 1 Instituto de Ciências da Atividade Física e Esportes, Universidade Cruzeiro do Sul, São Paulo, SP, Brazil; 2 Universidade Paulista, São Paulo, SP, Brazil; NYU Langone Medical Center, UNITED STATES

## Abstract

This study investigated the effects of palmitoleic acid on different phases of the healing process. Macroscopic analyses were performed on wounds in rats with or without palmitoleic acid treatment, and the results showed that palmitoleic acid directly hastened wound closure. The topical treatment of wounds with palmitoleic acid resulted in smaller wounds than those observed in the control group. The anti-inflammatory activity of palmitoleic acid may be responsible for healing, especially in the stages of granulation tissue formation and remodelling. Palmitoleic acid modified TNF-α, IL-1β, IL-6, CINC-2α/β, MIP-3α and VEGF-α profiles at the wound site 24, 48, 120, 216 and 288 hours post-wounding. Assays assessing neutrophil migration and exudate formation in sterile inflammatory air pouches revealed that palmitoleic acid had potent anti-inflammatory activity, inhibiting the LPS-induced release of TNF-α (73.14%, p≤0.05), IL-1β (66.19%, p≤0.001), IL-6 (75.19%, p≤0.001), MIP-3α (70.38%, p≤0.05), and l-selectin (16%, p≤0.05). Palmitoleic acid also inhibited LPS-stimulated neutrophil migration. We concluded that palmitoleic acid accelerates wound healing via an anti-inflammatory effect.

## Introduction

Palmitoleic acid, an abundant fatty acid found in plant oils from macadamia nuts, hazelnuts and sea-buckthorn, is found in the human skin, especially in young skin, and decreases with age. The high oxidative stability of macadamia oil, which is used in cosmetics, makes it particularly suitable for heavy creams and other pharmaceutical formulations [1]; however, little is known about its mechanisms of action and the effects of its components on wound healing. Recent studies have reported that palmitoleic acid is useful in treating disorders related to skin hyperpigmentation [2], fibrosis [3] and as an adjuvant in formulations for the treatment of secondary infections caused by gram-positive bacteria [4, 5, 6].

Cutaneous wound healing involves several components, such as platelets, resident cells (keratinocytes, fibroblasts, endothelial cells and nervous cells), leukocytes (macrophages, neutrophils and lymphocytes), lipid mediators such as prostanoids (prostaglandin, thromboxanes and prostacyclins), protein mediators (acute-phase proteins, cytokines and growth factors), and reactive oxygen and nitrogen species [7]. The healing process involves orchestrated phases (inflammation, new tissue formation and tissue remodelling) and may be modulated by fatty acids [8–12].

Because palmitoleic acid may affect immune cell functions and cytokines and growth factors are involved in the wound-healing process and fibrosis, we investigated the effects of palmitoleic acid at different phases of the healing process in rats. Based on *in vivo* assays, we performed kinetic macroscopic analyses of wound closure and closure velocity, and we determined the local concentrations of TNF-α, IL-1β, IL-6, CINC-2α/β, MIP-3α, l-selectin and VEGF-α in the wound at different phases and times (0, 4, 24, and 48 hours; 5, 9 and 12 days) of the healing process. The inflammatory phase of wound healing involves vascular and cellular events and is best characterized by neutrophil influx. These phagocytes regulate the initial phase of the healing process and orchestrate the subsequent phases. To investigate the effect of palmitoleic acid on the inflammatory phase of the healing process *in vivo*, we analysed the neutrophil influx into air pouches and the cytokines present in the exudate of rats treated or not treated with a pro-inflammatory stimulant (LPS) and/or palmitoleic acid.

## Materials and methods

### Animals

Adult male Wistar rats initially weighing 180±20 g were studied. The rats were obtained from the Institute of Biomedical Sciences (ICB), University of São Paulo (USP), São Paulo, Brazil. The rats were housed at 23°C in individual cages and under a light-dark cycle of 12:12 hours, and they were given *ad libitum* access to regular chow and water. The experimental procedure was approved by the Animal Care Committee of Cruzeiro do Sul University and was performed in agreement with the Guidelines for Ethical Conduct in the Care and Use of Animals in Research. After the end of experiments, the rats were euthanized by cervical displacement under anaesthesia, following protocol approved by the Ethics Committee.

### Induction and measurement of wound size

As previously described in detail by our group [12], the animals were initially anaesthetized using ketamine (60 mg/kg) and xylazine (10 mg/kg), after which a 10 mm^2^ piece of skin was surgically removed from the dorsal region of each rat. After surgery, the wound was treated topically with 100 μL of palmitoleic acid (100 μM) or the same volume of sterile phosphate-buffered saline (PBS solution, pH = 7.4). The rats were maintained in individual cages under a warming lamp and were monitored until fully recovered from the anaesthesia. The rats were divided into two groups: the control (PBS-treated) rats and the palmitoleic acid-treated (100 μM) rats. Palmitoleic acid or PBS was administered once daily. Based on the healing process time in rats and previous experiments by our group [10, 12], the 15th day was the end point of this experiment.

To evaluate wound closure, the wounds were photographed daily with a Nikon D7000 18–105 mm camera using the same focal length, lens aperture and exposure time. The photographs were digitized, and the wound area was measured using ImageJ software (National Institutes of Health, Bethesda, MD, USA). The total injury area and healing speed were monitored. Wound closure was defined as a reduction in the wound area, and the results were expressed as the percentage (%) of the original wound area [12].

### Air pouch assay and exudate collection and processing

As previously described in detail by Farsky et al. (1997) [13] and our group [10], rat skin air pouches were produced at the dorsal region of the animals to analyse neutrophil migration and measure cytokines. Initially, 20 mL of filtered sterile air (0.22 μm) was subcutaneously injected into the backs of anaesthetized rats. Seven days later, an additional 10 mL of sterile air was injected, and on the 8th day, 1 mL of a palmitoleic acid solution (100 μM) in sterile PBS was injected into the pouch under anaesthesia and aseptic conditions. The negative control animals received 1 mL of sterile PBS, and the positive controls received 1 mL of sterile PBS plus the inflammatory stimulus LPS (5 μg/mL) via the same route. Four hours after the palmitoleic acid treatment, the intraperitoneal cavity was washed with 10 mL of sterile PBS, and the inflammatory exudate was collected. The suspension was centrifuged at 500 g for 10 minutes at -4°C. Next, neutrophils were counted using a Neubauer chamber. We also measured cytokine levels in the supernatant by ELISA using a DuoSet kit (Quantikine DuoSet, R&D Systems, Minneapolis, MN, USA) [10].

### Cytokine levels in wounds

Wound tissues removed 0, 4, 24, 48, 120, 216 and 288 hours after surgery were immediately frozen (-80°C) until they were homogenized using PBS plus protease inhibitors (0.5 M PMSF and 25 IU mL^–1^ aprotinin). The tissue (100 mg) was homogenized in a Polytron PT 3100 homogenizer (Kinematica, Lucerne, Switzerland). TNF-α, IL-1β, CINC-2α/β, MIP-3, IL-6 and VEGF-α levels were assessed using ELISA (Quantikine DuoSet, R&D Systems, Minneapolis, MN, USA). The concentrations were normalized to the amount of protein in the samples, which was determined using the classical Bradford method.

### Statistical analysis

The statistical analysis was performed by comparing the control groups with the palmitoleic acid-treated groups. The groups were compared using ANOVA and the post hoc Student-Newman-Keuls multiple comparisons test and Dunnett’s test (InStat; GraphPad Software, San Diego, CA, USA). The significance level was set at p<0.05.

## Results

**Fig 1:**
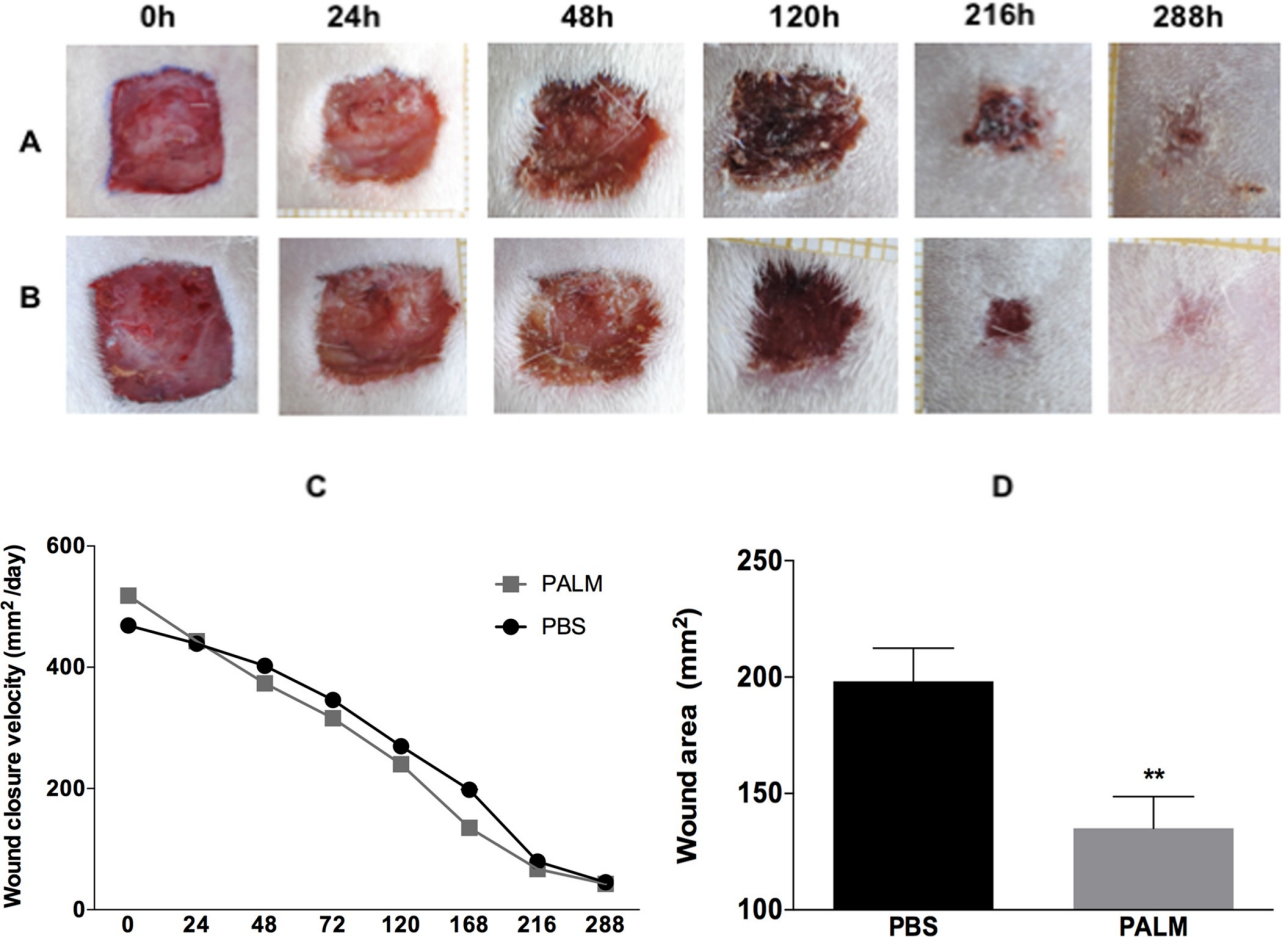
Wound closure velocity. Macroscopic wound closure in the control rats (A) and the rats treated daily with palmitoleic acid (B). Representative photos and wound area values recorded over a 12-day period. (C) Wound closure velocity (mm^2^/day) and (D) ratios of integrated wound closure area with or without palmitoleic treatment. Values are expressed as the mean±SEM of at least 10 animals per group. *p≤0.05 versus control, as indicated by analysis of variance (ANOVA) and post hoc Tukey’s test.

Photographic records were generated daily to analyse the macroscopic wound closure, total injury area and healing speeds of the control rats (Fig 1A) and those treated with palmitoleic acid (Fig 1B). As shown in Fig 1C, palmitoleic acid directly hastened the wound closure. Integration of curves representing the wound closure area monitored over 12 days demonstrated that treating the wound with palmitoleic acid decreased the wounded area compared with the untreated group (Fig 1D).

**Fig 2:**
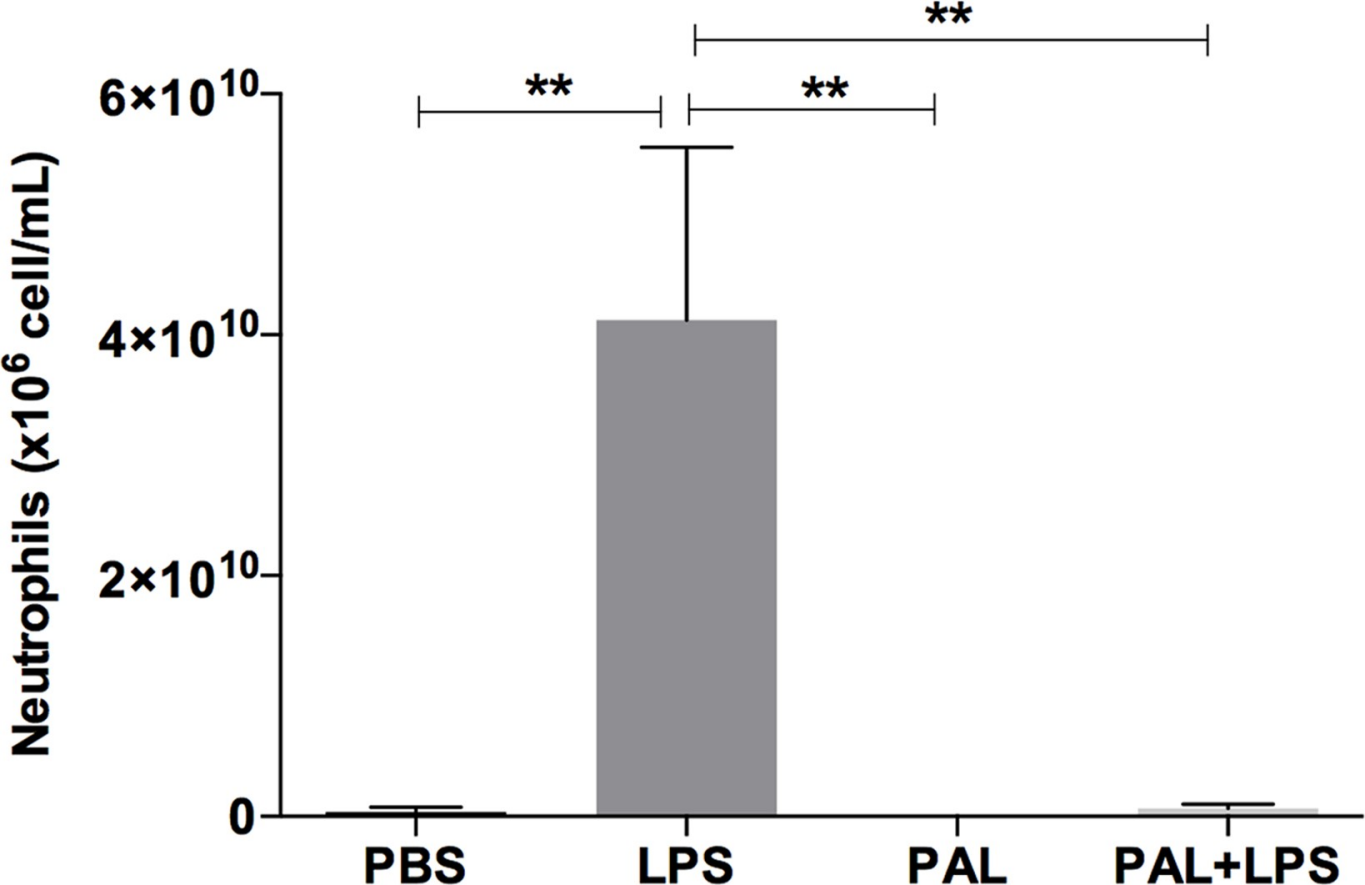
Neutrophils migration. Neutrophil influx into air pouches after palmitoleic acid (100 mM) injection. LPS (5 μg/mL) was used as a positive control. Values are presented as the means±SEM of eight animals per group. **p<0.01 for comparisons between treatments with fatty acids and control, as indicated.

The inflammatory phase of wound healing is characterized by increased neutrophil influx. This phase occurs in the first hours after injury. To investigate the effect of palmitoleic acid on the inflammatory phase of the wound-healing process *in vivo*, we analysed neutrophil influx into air pouches and the proteins in the exudate of rats treated or not with palmitoleic acid. The number of neutrophils that migrated to the air pouches was determined four hours after palmitoleic acid injection. LPS, a characteristic component of gram-negative bacterial cell walls that activates neutrophil movement to infected areas, induced significant neutrophilic influx into the pouches. Our results indicated that palmitoleic acid strongly inhibited LPS-stimulated neutrophil migration (Fig 2).

**Fig 3:**
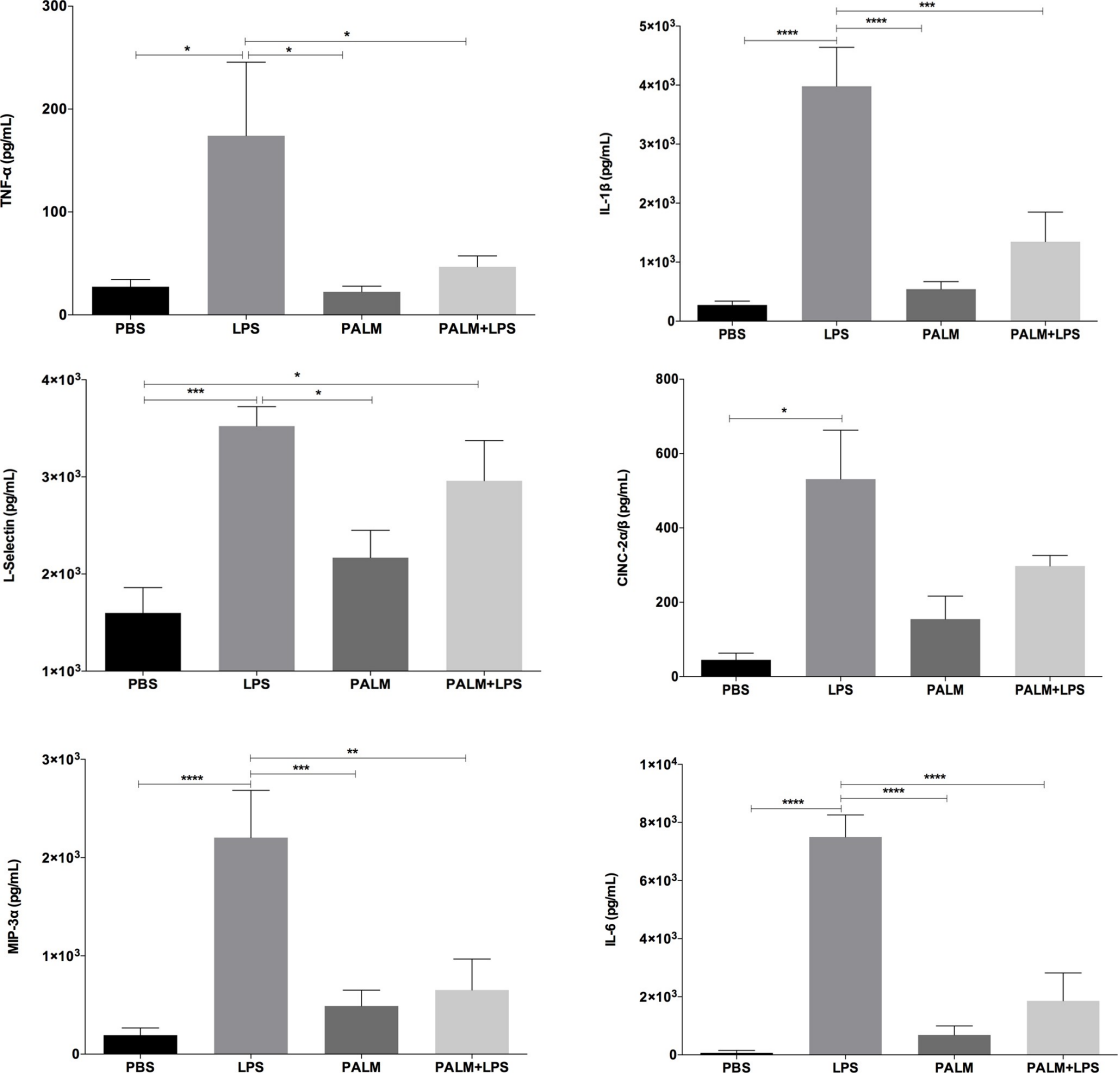
Air pouch cytokines release. Cytokine release into air pouches after palmitoleic acid (100 mM) injection. LPS (5 μg/mL) was used as a positive control. Values are presented as the means±SEM of eight animals per group. *p<0.05, **p<0.01 and ***p<0.001 for comparisons between treatments with fatty acids and control, as indicated by ANOVA and Dunnett’s test.

**Fig 4:**
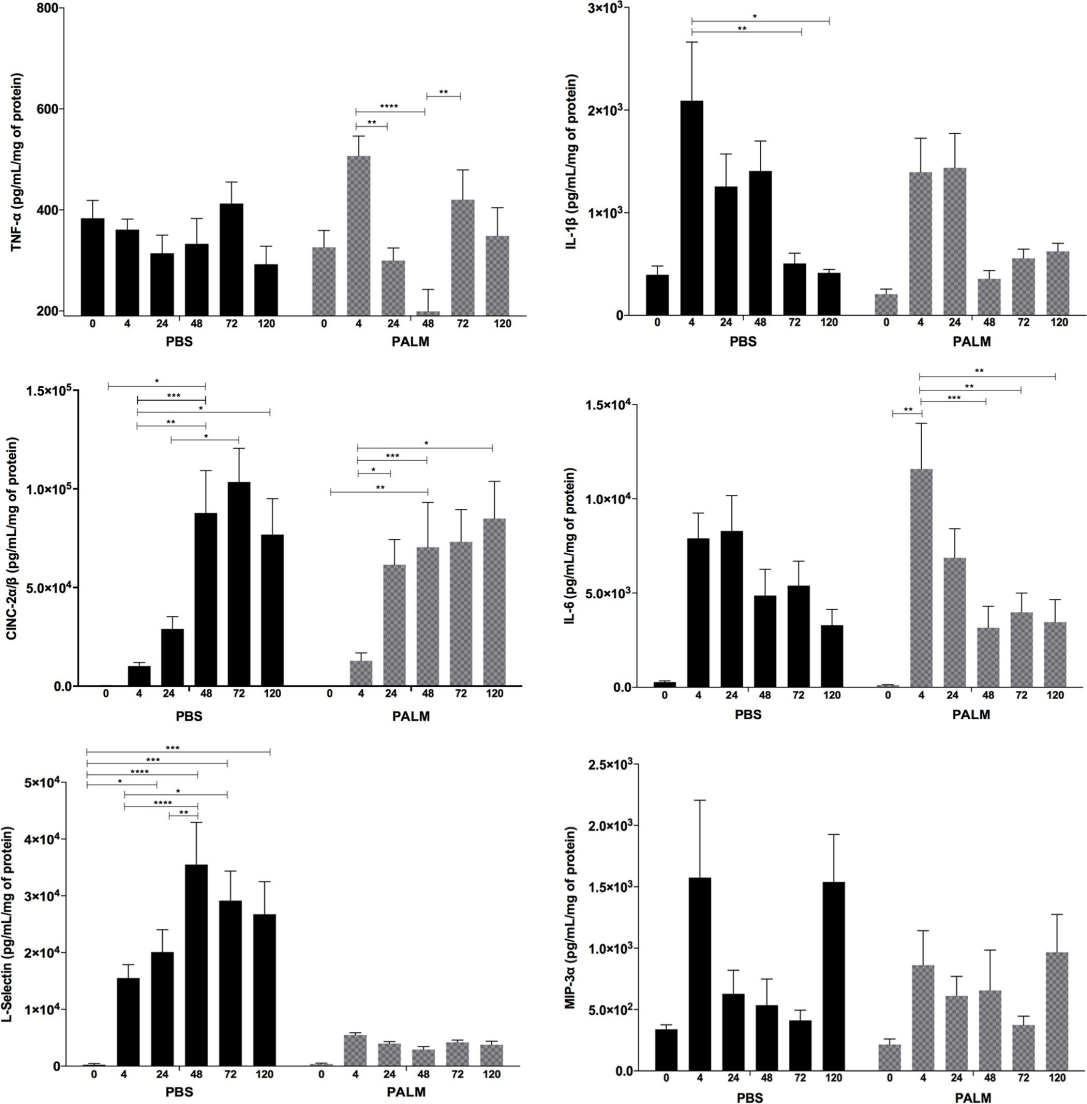
Cytokines concentration in wound. Kinetic profiles of cytokine concentrations in the control (PBS) and treated rats (PALM) measured before (0), 4 hours (4), 24 hours (24), 48 hours (48), 72 hours (72) and 120 hours (120) after wound induction. Values are presented as the mean±SE of at least 8 animals per group.

Neutrophils regulate the wound-healing process and orchestrate the inflammatory phase by migrating to the inflammatory focus; phagocytosing cellular debris and microorganisms; releasing pro-inflammatory cytokines, chemokines and angiogenic growth factors; and producing reactive oxygen species. These events are closely correlated and in some cases depend on signalling initiated by cytokines (TNF-α, IL-1β, and CINC-2α/β) and growth factors (VEGF-α). As Fig 3 indicates, the animals treated with palmitoleic acid showed markedly decreased LPS-induced inflammation. Palmitoleic acid had potent anti- inflammatory activity, inhibiting the LPS-induced release of TNF-α (73.14%, p≤0.05), IL-1β (66.19%, p≤0.001), IL-6 (75.19%, p≤0.001), MIP-3α (70.38%, p≤0.05), and l-selectin (16%, p≤0.05). VEGF release was unaltered (data not shown). Palmitoleic acid had potent anti- inflammatory activity. Concentrations of l-selectin, at wound site, was significantly lower after palmitoleic acid treatment in comparison with the control group (Fig 4).

## Discussion

Fatty acids can regulate the tissue regeneration process, which brings up several relevant points. For example, to optimize therapeutic interventions, prior knowledge of a drug’s effect on lesion chronology is necessary to maximize the treatment benefits. Based on the present analysis, we intend to further study fatty acids and choose the ideal intervention to maximize the predominant event in each phase of the healing process. For example, administering boosters for neutrophil migration would be pointless if the lesion chronology was to indicate a predominance of epithelialization. Likewise, administering keratinocyte migration stimulators would not affect the initial injury phase.

To develop new therapeutic agents, it is essential to elucidate the mechanisms whereby specific fatty acids act to repair tissue. Although many studies have focused on fatty acids and their functions in immune cells [14–20], few studies have addressed the topical effects of fatty acids on signalling in the healing process. Most studies have addressed topical wound treatment with oleic and linoleic acid or fatty acid intake.

In recent years, our group has focused on elucidating the functions of different fatty acids in the healing process [10–12] and the cells involved in tissue repair, such as neutrophils [14, 16–18], macrophages [20] and fibroblasts [19, 21].

Promising studies have demonstrated multiple functions of fatty acids in the epidermis, in addition to their primary functions as an energy source, in storage, and in the membrane lipid bilayer [22–24]. Fatty acids are important in the formation of the permeability barrier, which contributes to the acidification of the stratum corneum, promoting its structural integrity and barrier function [25]. These acids also serve as building blocks for complex lipids in the sebum produced by sebaceous glands [26]. Sebum lipids impart a self-disinfecting activity to the skin surface, and free fatty acids are responsible for this property [26]. Studies by Cardoso et al. (2004, 2011) [8, 9] and Pereira et al. (2008) [10] have demonstrated that topical wound treatment with oleic and linoleic acids accelerates tissue repair mechanisms due to the ability of these fatty acids to modulate inflammation. Oleic and linoleic acid treatment increased neutrophils in the wound and reduced the necrotic layer thickness [10]. Incubating neutrophils with oleic and linoleic acids dose-dependently increased the release of IL-1β and VEGF-α. These effects of oleic and linoleic acids are important in situations of neutrophil dysfunction and non-healing wounds, such as diabetes [10]. Additionally, the oily, moist dressings that characterize fatty acid applications serve as protective barriers against microorganisms, prevent tissue dehydration, and maintain decreases in body temperature during skin replacement in trauma healing [27]. Autolysis, the natural degradation of devitalized tissue through the actions of enzymes such as acid hydrolases, is favoured in wounds treated with wet dressings. Other advantages of keeping wounds hydrated include the stimulation of epithelialization, the formation of granulation tissue and angiogenesis [28].

Bioactive compounds, such as fatty acids, show a protective effect against stress-induced senescence in the skin and under conditions that may lead to the development of senescence, such as UV-A and UV-B irradiation of cells and the production of matrix metalloproteinases [29]. The addition of palmitoleic acid isomers produced antibacterial activity and bactericidal properties [5, 6].

In this study, we found that palmitoleic acid had anti-inflammatory effects, decreasing inflammation. Topical fatty acids are used successfully to treat open injuries in humans, with or without infection, especially in Latin America [27].

Diabetes and obesity are examples of conditions that contribute to chronic wounds worldwide. Many cost-effective wound-healing technologies have recently been a focus of research due to increased demand [30, 31]. Our results demonstrate that palmitoleic acid directly hastens wound closure. The factor responsible for this healing may be the anti-inflammatory action of palmitoleic acid; however, it is important to note that the underlying mechanisms of palmitoleic acid-induced changes in wound closure remain unknown and lie outside the scope of this study.

## References

[1] Kolouchová I, Sigler K, Schreiberová O, Masák J, Řezanka T. New yeast-based approaches in production of palmitoleic acid. Bioresour Technol. 2015;192: 726–34. 10.1016/j.biortech.2015.06.048 26101962

[2] Yoon WJ, Kim MJ, Moon HJ, Kim GO, Lee NH, Hyun CG. Effect of palmitoleic acid on melanogenic protein expression in murine b16 melanoma. J Oleo Sci 59:315–319, 2010 20484837 10.5650/jos.59.315

[3] Fischer CL, Drake DR, Dawson DV, Blanchette DR, Brogden KA, Wertz PW. Antibacterial activity of sphingoid bases and fatty acods against Gram-positive and Gram-negative bacteria. Antimicrob Agents Chemother 56:1157–1161, 2012 10.1128/AAC.05151-11 22155833 PMC3294957

[4] Wille JJ, Kydonieus A. Palmitoleic acid isomer (C16:1delta6) in human skin sebum is effective againstgram-positive bacteria. Skin Pharmacol Appl Skin Physiol. 2003;16:176–187. 10.1159/000069757 12677098

[5] Gao ZL, Gu XH, Cheng FT, Jiang FH Sea buckthorn may be a hopeful drug for prevention and treatment of liver fibrosis. Effect of Sea buckthorn on liver fibrosis: A clinical study. World J Gastroenterol. 2003; 9: 1615–1617. 10.3748/wjg.v9.i7.1615 12854177 PMC4615518

[6] Akamatsu H, Oguchi M, Nishijima S, Asada Y, Takahashi M, Ushijima T, Niwa Y. The inhibition of free radical generation by human neutrophils through the synergistic effects of metronidazole with palmitoleic acid: a possible mechanism of action of metronidazole in rosacea and acne. Arch Dermatol Res. 1990;282: 449–454. 2150301 10.1007/BF00402621

[7] Gurtner GC, Werner S, Barrandon Y, Longaker MT. Wound repair and regeneration. Nature. 2008;453: 314–321. 10.1038/nature07039 18480812

[8] Cardoso CR, Souza MA, Ferro EA, Favoreto S Jr, Pena JD. Influence of topicaladministration of n-3 and n-6 essential and n-9 nonessential fatty acids on the healing of cutaneous wounds. Wound Repair Regen. 2004;12: 235–243. 10.1111/j.1067-1927.2004.012216.x 15086775

[9] Cardoso CR, Favoreto S Jr, Oliveira LL, Vancim JO, Barban GB, Ferraz DB, Silva JS. Oleic acid modulation of the immune response in wound healing: a new approach for skin repair. Immunobiology. 2011; 216: 409–415. 10.1016/j.imbio.2010.06.007 20655616

[10] Pereira LM, Hatanaka E, Martins EF, Oliveira F, Liberti EA, Farsky SH, Curi R, Pithon-Curi TC. Effect of oleic and linoleic acids on the inflammatory phase of wound healing in rats. Cell Biochem Funct. 2008;26:197–204. 10.1002/cbf.1432 17918246

[11] Rodrigues HG, Vinolo MA, Magdalon J, Fujiwara H, Cavalcanti DM, Farsky SH, Calder PC, Hatanaka E, Curi R. Dietary free oleic and linoleic acid enhances neutrophil function and modulates the inflammatory response in rats. Lipids. 2010;45: 809–819. 10.1007/s11745-010-3461-9 20730605

[12] Rodrigues HG, Vinolo MA, Magdalon J, Vitzel K, Nachbar RT, Pessoa AF, dos Santos MF, Hatanaka E, Calder PC, Curi R. Oral administration of oleic or linoleic acid accelerates the inflammatory phase of wound healing. J Invest Dermatol. 2012;132:208–215. 10.1038/jid.2011.265 21881592

[13] Farsky SH, Walber J, Costa-Cruz M, Cury Y, Teixeira CF, Curry Y. Leukocyte response induced by Bothrops jararaca crude venom: in vivo and in vitro studies. Toxicon 1997;35: 185–193. 9080575 10.1016/s0041-0101(96)00135-3

[14] Hatanaka E, Levada-Pires AC, Pithon-Curi TC, Curi R. Systematic study on ROS production induced by oleic, linoleic, and gamma-linolenic acids in human and rat neutrophils. Free Radic Biol Med. 2006;41:1124–1132. 10.1016/j.freeradbiomed.2006.06.014 16962937

[15] Martins de Lima T, Gorjão R, Hatanaka E, Cury-Boaventura MF, Portioli Silva EP, Procopio J, Curi R. Mechanisms by which fatty acids regulate leucocyte function. Clin Sci. 2007;113(2):65–77. 10.1042/CS20070006 17555405

[16] Vinolo MA, Hatanaka E, Lambertucci RH, Newsholme P, Curi R. Effects of short chain fatty acids on effector mechanisms of neutrophils. Cell Biochem Funct. 2009;27: 48–55. 10.1002/cbf.1533 19107872

[17] Vinolo MA, Rodrigues HG, Hatanaka E, Hebeda CB, Farsky SH, Curi R. Short-chain fatty acids stimulate the migration of neutrophils to inflammatory sites. Clin Sci. 2009;117:331–338. 10.1042/CS20080642 19335337

[18] Vinolo MA, Rodrigues HG, Hatanaka E, Sato FT, Sampaio SC, Curi R. Suppressive effect of short-chain fatty acids on production of proinflammatory mediators by neutrophils. J Nutr Biochem. 2011;22:849–855. 10.1016/j.jnutbio.2010.07.009 21167700

[19] Magdalon J, Hatanaka E, Romanatto T, Rodrigues HG, Kuwabara WM, Scaife C, Newsholme P, Curi R. A proteomic analysis of the functional effects of fatty acids in NIH 3T3 fibroblasts. Lipids Health Dis. 2011 24;10:218 10.1186/1476-511X-10-218 22114894 PMC3281802

[20] Magdalon J, Vinolo MA, Rodrigues HG, Paschoal VA, Torres RP, Mancini-Filho J, Calder PC, Hatanaka E, Curi R. Oral administration of oleic or linoleic acids modulates the production of inflammatory mediators by rat macrophages. Lipids. 2012;47:803–812. 10.1007/s11745-012-3687-9 22695743

[21] Hatanaka E, Dermargos A, Hirata AE, Vinolo MA, Carpinelli AR, Newsholme P, Armelin HA, Curi R. Oleic, linoleic and linolenic acids increase ros production by fibroblasts via NADPH oxidase activation. PLoS One. 2013 4 8;8(4):e58626 10.1371/journal.pone.0058626 23579616 PMC3620266

[22] Ruthig DJ, Meckling-Gill KA. Both (n-3) and (n-6) fatty acids stimulate wound healing in the rat intestinal epithelial cell line, IEC-6. J Nutr. 1999;129:1791–1798. 10.1093/jn/129.10.1791 10498749

[23] Calder PC. n-3 fatty acids, inflammation, and immunity-relevance to postsurgical and critically ill patients. Lipids. 2004;39: 1147–1161. 15736910 10.1007/s11745-004-1342-z PMC7101959

[24] Calder PC. Long-chain n-3 fatty acids and inflammation: potential application in surgical and trauma patients. Brasilian Journal of Medical and Biological Research 2003; 36: 433–446. 10.1590/s0100-879x2003000400004 12700820

[25] Ziboh VA, Miller CC, Cho Y. Metabolism of polyunsaturated fatty acids by skin epidermal enzymes: generation of antiinflammatory and antiproliferative metabolites. Am J Clin Nutr 71:361S–6S, 2000 10.1093/ajcn/71.1.361s 10617998

[26] Nakatsuji T, Kao MC, Zhang L, Zouboulis CC, Gallo RL, Huang CM. Sebum free fatty acids enhance the innate immune defense of human sebocytes by upregulating beta-defensin-2 expression. J Invest Dermatol. 2010;130:985–994. 10.1038/jid.2009.384 20032992 PMC3057125

[27] Pieper B, Caliri MH. Nontraditional wound care: A review of the evidence for the use of sugar, papaya/papain, and fatty acids. J Wound Ostomy Continence Nurs. 2003; 30:175–183. 10.1067/mjw.2003.131 12851592

[28] Svensjö T, Pomahac B, Yao F, Slama J, Eriksson E. Accelerated healing of full-thickness skin wounds in a wet environment. Plast Reconstr Surg. 2000;106:602–614. 10987467

[29] Mária J, Ingrid Ž. Effects of bioactive compounds on senescence and components of senescence associated secretory phenotypes in vitro. Food Funct. 2017;8:2394–2418. 10.1039/c7fo00161d 28665427

[30] Gwak Jae Ha, Sohn So Young. Identifying the trends in wound-healing patents for successful investment strategies PLoS One. 2017; 12(3): e0174203 10.1371/journal.pone.0174203 28306732 PMC5357059

[31] Lima MHM, Caricilli AM, Abreu LL, Araújo EP, Pelegrinelli FF, Thirone ACP, Tsukumo DM, Pessoa AFM, dos Santos MF, de Moraes M A, Carvalheira JBC, Velloso LA, Saad MJA. Topical Insulin Accelerates Wound Healing in Diabetes by Enhancing the AKT and ERK Pathways: A Double-Blind Placebo-Controlled Clinical Trial. PLoS One. 2012; 7(5): e36974 10.1371/journal.pone.0036974 22662132 PMC3360697

